# Investigation of Genetic Disturbances in Oxygen Sensing and Erythropoietin Signaling Pathways in Cases of Idiopathic Erythrocytosis

**DOI:** 10.1155/2013/495724

**Published:** 2013-12-02

**Authors:** Carla Luana Dinardo, Paulo Caleb Junior Lima Santos, Isolmar Tadeu Schettert, Renata Alonso Gadi Soares, Jose Eduardo Krieger, Alexandre Costa Pereira

**Affiliations:** ^1^Laboratory of Genetics and Molecular Cardiology, Heart Institute, University of São Paulo Medical School, Avenue Dr. Enéas de Carvalho Aguiar, 44 Cerqueira César, 05403-000 São Paulo, SP, Brazil; ^2^Novo Atibaia Hospital, 05403-000 São Paulo, SP, Brazil

## Abstract

*Background*. Idiopathic erythrocytosis is the term reserved for cases with unexplained origins of abnormally increased hemoglobin after initial investigation. Extensive molecular investigation of genes associated with oxygen sensing and erythropoietin signaling pathways, in those cases, usually involves sequencing all of their exons and it may be time consuming. *Aim*. To perform a strategy for molecular investigation of patients with idiopathic erythrocytosis regarding oxygen sensing and erythropoietin signaling pathways. *Methods*. Samples of patients with idiopathic erythrocytosis were evaluated for the *EPOR, VHL, PHD2*, and *HIF-2**α*** genes using bidirectional sequencing of their hotspots. *Results*. One case was associated with *HIF-2**α*** mutation. Sequencing did not identify any pathogenic mutation in 4 of 5 cases studied in any of the studied genes. Three known nonpathogenic polymorphisms were found (*VHL* p.P25L, rs35460768; *HIF-2**α*** p.N636N, rs35606117; *HIF-2**α*** p.P579P, rs184760160). *Conclusion*. Extensive molecular investigation of cases considered as idiopathic erythrocytosis does not frequently change the treatment of the patient. However, we propose a complementary molecular investigation of those cases comprising genes associated with erythrocytosis phenotype to meet both academic and genetic counseling purposes.

## 1. Introduction

**Figure 1:**
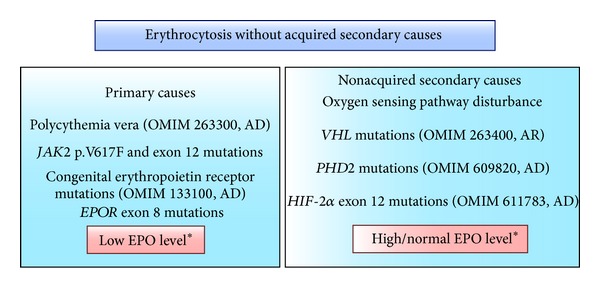
Representation of diagnostic strategy for patients with erythrocytosis without acquired secondary causes. The most common example of primary erythrocytosis is polycythemia vera (PV). Notably, the majority of idiopathic erythrocytosis patients cannot be classified into the OMIM categories, nor do they have a characterized molecular defect. Consequently, patients with established but unexplained erythrocytosis warrant further investigation [1]. AD: autosomal dominant inheritance, AR: autosomal recessive inheritance. *EPO levels can suggest origins of this disorder.

Absolute erythrocytosis should be suspected when hemoglobin (Hb) is greater than 16.5 g/dL in females or 18.0 g/dL in males [1] and defines a status of elevated hemoglobin. Causes of erythrocytosis can be divided into two groups: primary, in which there is an intrinsic problem in bone marrow driving abnormal erythropoiesis, or secondary, in which there is an event outside bone marrow leading to an abnormal production of erythrocytes [1]. Both groups can be further classified into acquired or congenital. Within the group of primary erythrocytosis, erythropoietin receptor (EPOR) mutation (congenital) and polycythemia vera (PV, acquired) stand out. Within the group of secondary erythrocytosis, there are multiple acquired causes (hypoxia, EPO pathologic production, and drugs) and a restricted subgroup of congenital causes (*VHL*, *PHD2*, and *HIF-2*α** mutations; high oxygen-affinity hemoglobin; and bisphosphoglycerate mutase deficiency) (Figure 1) [1–3].

Idiopathic erythrocytosis is the term reserved for the cases with an unexplained cause of erythrocytosis after initial investigation. Even though this classification is currently applied in clinical practice, it lacks precise criteria. Usually, a patient that neither could be classified as PV nor presents with an acquired cause of erythrocytosis is labeled as having idiopathic erythrocytosis. Further investigation is of questionable clinical value. Cases with a suspicion of congenital causes may be associated with disturbances of oxygen sensing and erythropoietin signaling pathways.

In this scenario, we propose a molecular strategy for evaluating genes regarding oxygen sensing and erythropoietin signaling pathways in cases of idiopathic erythrocytosis with suspected congenital causes.

## 2. Methods

Five Brazilian patients with suspected idiopathic erythrocytosis were selected at Novo Atibaia Hospital, SP, Brazil, and Heart Institute, SP, Brazil. The study protocol was approved by the involved Institutional Ethics Committees, and written informed consent was obtained from all patients. General characteristics of patients were obtained through an interview.

Patients enrolled fulfilled the following inclusion criteria:

1. documented persistent erythrocytosis (16.5 g/dL in females or 18.0 g/dL in males);
2. *JAK2* p.V617F negative and normal *JAK2* exon 12 sequencing;
3. arterial blood gas analysis (including p50) within normal range;
4. biochemical analysis within normal range;
5. bone marrow biopsy normal or revealing erythroid expansion;
6. absence of symptoms of obstructive sleep apnea or normal polysomnography;
7. absence of masses in abdominal tomography or abdominal ultrasound.

Genomic DNA was isolated from peripheral blood leukocytes by a salting-out method. Coding sequences of *EPOR* exon 8,* VHL* exons 1–3, *PHD2* exons 1–5, and *HIF-2*α** exon 12 were amplified by a polymerase chain reaction (PCR) using the previously described primer sequences [4, 5]. Exons were selected based on literature review and consisted of known hotspots. PCR products were purified using ExoSAP-IT reagent (GE Healthcare, NJ, USA) and were bidirectionally sequenced (ABI Terminator Sequencing Kit and ABI 3500XL Sequencer, Applied Biosystems, Foster City, CA, USA).

## 3. Results and Discussion

We identified a novel mutation in *HIF-2*α** exon 12 in one patient, using the suggested investigation, which was published elsewhere and comprised a strong family history of arterial thromboembolic events [6]. The others had EPO levels within normal range and absence of family history of erythrocytosis or thromboembolism and sequencing of *EPOR* exon 8,* VHL *exons 1–3, *PHD2* exons 1–5, and *HIF-2*α** exon 12 did not identify any mutation. Three known nonpathogenic polymorphisms were found: *VHL* c.C74T polymorphism (p.P25L, rs35460768, exon 1, and heterozygous genotype), *HIF-2*α** c.T1908C polymorphism (p.N636N, rs35606117, exon 12, and heterozygous genotype), and *HIF-2*α** c.G1737A polymorphism (p.P579P, rs184760160, exon 12, and heterozygous genotype). Results were ready after less than one week after blood samples were obtained. Phlebotomies and acetylsalicylic acid use (100 mg/day) were prescribed to all patients, with normalization of hemoglobin and hematocrit values and favorable outcome.

The investigation of a patient with absolute erythrocytosis involves a detailed clinical history and physical examination as well as an already standardized laboratory evaluation. In cases of absolute erythrocytosis without any acquired secondary cause, *PV* should be ruled out at first and considering the current dissemination of molecular techniques designed to detect p.*V617F JAK2* mutation, most medical centers manage to diagnose their *PV* cases without great difficulties. Sequencing *JAK2* exon 12, the next step if *JAK2* p.V617F mutation is absent, is quite more challenging and easily forgotten during the laboratory investigation [7]. Besides *JAK2* exon sequencing, detection of mutation on this region can be also performed for high resolution melting (HRM) analysis as shown by previous studies [8–10].

Many physicians would not go further after this initial investigation, since there will be no significant changes regarding patients' clinical management. However, when there is a suspicion of a congenital cause behind a case of idiopathic erythrocytosis, a complementary molecular investigation regarding the most important causes may become important, especially in terms of genetic family counseling. According to current medical literature, disturbances involving oxygen sensing pathway, presence of high oxygen affinity hemoglobin, and mutations in erythropoietin receptor (EPOR) stand out in the group of congenital causes of erythrocytosis.

In order to avoid extensive sequencing strategies comprising all exons of all genes involved in oxygen sensing and erythropoietin signaling pathways, we validated in this study a protocol in which the most important genes of both pathways are evaluated and, in those two chosen genes, only hotspots exons are sequenced [5, 11–13]. Our protocol consists in sequencing *EPOR* exon 8, *VHL* exons 1–3, *PHD2* exons 1–5, and *HIF2-*α** exon 12. For a more structured investigation, we suggest sequencing *EPOR* exon 8 for cases of idiopathic erythrocytosis where EPO level is low and sequencing *VHL* exons 1–3, *PHD2* exons 1–5, and *HIF2-*α** exon 12 for cases of idiopathic erythrocytosis where EPO level is normal or high (Figure 1).

In our presented validation, family history of erythrocytosis/arterial thromboembolic events was the most important anamnesis data, as it was absent in the cases where no mutations were found but present in the mutant case. One drawback of our suggested sequencing strategy is that some nonpathogenic polymorphisms may be identified, but bioinformatics programs can easily overcome this obstacle. A comprehensive database concerning molecular and laboratory data of congenital erythrocytosis patients is provided by the EU Congenital Erythrocytosis (ECE-C) Consortium (http://www.erythrocytosis.org). In addition, it represents a useful tool when evaluating if a certain discovered genetic alteration has already been described as pathogenic [14, 15]. Another main database about myeloproliferative diseases is the European Cooperation in Science and Technology (http://www.mpneuronet.eu). An available reference, “Clinical utility gene card for familial erythrocytosis,” shows genetic, clinical, and practical aspects about erythrocytosis [16]. Studies indicated an undetermined proportion of positive genetic tests if the disease is present. But, they suggest that <50% of cases with erythrocytosis and positive family history are likely positive for one of the associated mutations [1, 16].

The ECE-C has recently described their strategy for evaluating patients with congenital erythrocytosis [15]. Our suggested strategy is similar to theirs in the case of CE patients with low EPO level, as sequencing *EPOR* exon 8 is suggested. In the case of CE patients with normal or high EPO level, the ECE-C organizes their sequencing strategy based on the presence or absence of family history. In our guideline, we suggested sequencing *VHL* exons 1–3, *PHD2* exons 1–5, and *HIF2-*α** exon 12 irrespective of family history, mainly because of the high prevalence of cardiovascular diseases, which hampers the distinction between real/casual antecedents and may impair the investigation and also because of the logistics facility of performing all sequencing reactions together, considering the low amount of samples. 

In our suggested investigation strategy, we do not consider routinely sequencing other less commonly affected genes when the initial approach does not give a positive result. This is based on the fact that our CE patients with a negative investigation presented a favorable outcome after the beginning of the phlebotomy routine and also on our aims of providing a rational, cost-effective, and non-time-consuming tool. However, when a mutation is found, patients can be properly followed and evaluated regarding complications that may not be directly related to the level of hemoglobin, as it has already been previously described for mutation in *HIF* gene [17] and for the *VHL* gene [18, 19].

Nowadays, to perform next generation sequencing (NGS) for new candidate genes poses a promising tool to discover new mutations in CE patients. However, considering the costs and time consumption of this strategy, it should still be reserved for only some centers or academic purposes. New genes and mutations discovered using this technology should be evaluated regarding pathogenicity to, then, be incorporated to erythrocytosis-related genes group. In the cases with strong family history, to expand investigation and eventually to perform NGS may be advised, as the odd of discovering new mutations is higher.

## 4. Conclusion

We propose the investigation of disturbances of oxygen sensing and erythropoietin signaling pathways in cases of idiopathic erythrocytosis with positive family history of erythrocytosis/thromboembolism. Our suggested sequencing protocol is economically feasible and sensitive, and it might help both academic and genetic counseling purposes.
